# Elevated CHI3L1 as a Potential Biomarker of Cognitive Dysfunction in Anti‐NMDAR Encephalitis: Evidence From Clinical Results and Mice Model

**DOI:** 10.1002/cns.70739

**Published:** 2026-01-05

**Authors:** Yuhang Li, Ran Ding, Jiaxin Yang, Xiaoyue Yang, Ziyao Han, Xue Li, Jie Liu, Yan Jiang, Li Cheng, Jiannan Ma, Hanyu Luo, Li Jiang

**Affiliations:** ^1^ Department of Neurology, National Clinical Research Center for Children and Adolescents‘ Health and Diseases, Ministry of Education Key Laboratory of Child Development and Disorders, Chongqing Key Laboratory of Child Neurodevelopment and Cognitive Disorders Children‘s Hospital of Chongqing Medical University Chongqing China; ^2^ Department of Psychiatry The First Affiliated Hospital of Wenzhou Medical University Wenzhou China

**Keywords:** anti‐NMDAR encephalitis, astrocytes, CHI3L1, cognitive dysfunction, neurogenesis

## Abstract

**Background:**

Anti‐N‐methyl‐D‐aspartate receptor (NMDAR) encephalitis is frequently associated with long‐term cognitive impairment. However, the underlying mechanisms remain poorly understood, and reliable biomarkers for predicting cognitive outcomes are lacking.

**Methods:**

We established an active immunization mouse model of anti‐NMDAR encephalitis by immunizing with the GluN1_356‐385_ peptide. Hippocampal proteomic profiling was performed, followed by molecular and histological validation. Behavioral tests were used to assess cognitive function. In parallel, serum and cerebrospinal fluid (CSF) samples were analyzed from a clinical cohort of children with anti‐NMDAR encephalitis to evaluate expression of the targeted biomarker and its association with clinical outcomes.

**Results:**

Proteomic analysis and subsequent validation revealed significant upregulation of chitinase‐3‐like protein 1 (CHI3L1) in the hippocampus of model mice, primarily derived from astrocytes. Elevated CHI3L1 levels were observed in parallel with impaired hippocampal neurogenesis, reflected by decreased DCX^+^ immature neurons and increased SOX2^+^ neural progenitors. These changes were accompanied by cognitive deficits in behavioral tests. In parallel, we analyzed a pediatric cohort of 83 children with anti‐NMDAR encephalitis. CHI3L1 levels in both serum and CSF were significantly elevated compared to controls. While CHI3L1 levels showed no association with modified Rankin Scale scores at one‐year follow‐up, higher CHI3L1 levels in serum and CSF were significantly correlated with persistent cognitive impairment.

**Conclusions:**

Our findings provide preliminary evidence that astrocyte‐derived CHI3L1 may contribute to disrupted hippocampal neurogenesis and cognitive dysfunction in anti‐NMDAR encephalitis. CHI3L1 may serve as a potential biomarker for cognitive prognosis and a therapeutic target for reducing long‐term neurological sequelae.

## Graphical abstract

Astrocyte‐derived CHI3L1 is elevated in both a mouse model and pediatric patients with anti‐NMDAR encephalitis, coinciding with impaired hippocampal neurogenesis and cognitive deficits. CHI3L1 may serve as a potential biomarker for long‐term cognitive dysfunction in anti‐NMDAR encephalitis.

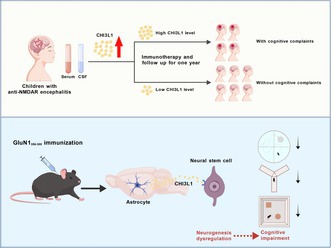

**Abbreviations**
CFA: Complete Freund's Adjuvant
CHI3L1: Chitinase‐3‐like protein 1
CNS: Central Nervous System
CSF: Cerebrospinal Fluids
DEP: Differentially Expressed Protein
mRS: modified Rankin Scale
NMDAR: N‐methyl‐D‐aspartate receptor
NSC: Neural Stem Cell

## 1 Introduction

Anti‐N‐methyl‐D‐aspartate receptor (NMDAR) encephalitis is the most common form of autoimmune encephalitis [1, 2]. It is characterized by a wide range of acute‐phase symptoms, including psychiatric disturbances, seizures, movement disorders, and decreased consciousness [3, 4], primarily resulting from autoantibody‐mediated internalization of synaptic NMDARs [5, 6]. Although immunotherapy is often effective in controlling acute symptoms, a substantial proportion of patients continue to experience persistent cognitive deficits—particularly in memory and executive functions—even after the clearance of pathogenic antibodies [7, 8, 9, 10]. While children with anti‐NMDAR encephalitis are generally considered to have more favorable prognoses than adults, several studies have suggested that pediatric patients may, in fact, exhibit more pronounced cognitive impairments, which can significantly hinder their learning, development, and quality of life [11, 12, 13, 14, 15, 16]. However, the mechanisms underlying these long‐term neurological sequelae remain largely unknown, and there is a critical lack of reliable biomarkers or therapeutic targets to monitor or intervene in this process.

Chitinase‐3‐like protein 1 (CHI3L1), also known as YKL‐40, is a glycoprotein predominantly secreted by reactive astrocytes in the central nervous system (CNS) under inflammatory conditions [17]. Elevated CHI3L1 levels have been reported in various neuroinflammatory and neurodegenerative disorders, including multiple sclerosis [18], neuromyelitis optica [19], Alzheimer's disease [20], and Parkinson's disease [21], where it is closely associated with glial activation and tissue remodeling [22]. Two previous studies have shown that cerebrospinal fluid (CSF) CHI3L1 levels are significantly increased in patients with anti‐NMDAR encephalitis compared to controls [23, 24]. However, whether this elevation is associated with poor neurological outcomes and what specific role CHI3L1 plays in the pathogenesis of this disease remains largely unexplored.

In this study, we first investigated the expression pattern and potential biological significance of CHI3L1 in anti‐NMDAR encephalitis using an active immunization mouse model. We then validated the changes in CHI3L1 levels in serum and CSF, as well as their association with clinical outcomes, in a large pediatric cohort with anti‐NMDAR encephalitis. Our findings suggest that CHI3L1 may contribute to the pathophysiology of anti‐NMDAR encephalitis through astrocyte‐mediated neuroinflammation and impaired neurogenesis, providing new insights into potential biomarkers and therapeutic targets for this devastating disease.

## 2 Methods and Materials

### 2.1 Patients and Samples

Children who met the current diagnostic criteria for anti‐NMDAR encephalitis were prospectively enrolled [25]. CSF and serum samples were collected prior to immunotherapy and stored at −80°C. Age‐matched samples from children with non‐inflammatory CNS diseases were used as controls. Detailed clinical information and outcomes were obtained from medical records and routine follow‐up visits. A modified Rankin Scale (mRS) score > 2 at 1 year after disease onset was defined as a poor outcome. In addition to the mRS assessment, patients and their parents were also interviewed regarding subjective cognitive complaints, including difficulties in learning, memory impairment, decreased attention, executive dysfunction, and related aspects. Patient recruitment and sample collection were approved by the Institutional Review Board of the Children's Hospital of Chongqing Medical University (Approval No. 2024344).

### 2.2 Cell Culture and Collection of Culture Supernatants

The human microglial cell line HMC3 (C5079, BaiDi Biotechnology Co. Ltd., China) and the human astrocyte cell line SVG p12 (YC‐D056, Ubigene Biosciences, Guangzhou, China) were maintained in DMEM (L110KJ, BasalMedia Technologies, Shanghai, China) supplemented with 10% fetal bovine serum (10099141C, Gibco) and 1% penicillin–streptomycin (ST488S, Beyotime, Shanghai, China). Cells were cultured at 37°C in a humidified incubator with 5% CO_2_ and were passaged using trypsin–EDTA (25200056, Gibco) upon reaching 80%–90% confluence.

To investigate the cellular origin of CHI3L1, HMC3 microglia and SVG p12 astrocytes were stimulated with pooled CSF obtained from patients with anti‐NMDAR encephalitis or from controls for 24 h. Culture supernatants were then collected and stored for subsequent CHI3L1 quantification.

### 2.3 Measurement of CHI3L1 Levels

CHI3L1 levels in CSF, serum, and supernatants were measured using a commercial ELISA kit (JL14809, JONLNBIO, Shanghai, China) according to the manufacturer's instructions. All samples were tested in duplicate.

### 2.4 Mice and Establishment Anti‐NMDAR Encephalitis Model

Female C57BL/6J mice aged 6 to 8 weeks were obtained from the Laboratory Animal Center of Chongqing Medical University. The mice were maintained under standardized environmental conditions, including a 12‐h light/dark cycle, ambient temperatures of 22°C–24°C, and relative humidity of 50%–60%. Food and water were freely available throughout the experiment. All animal protocols were approved by the Ethics Committee of the Children's Hospital of Chongqing Medical University (Approval No. CHCMU‐IACUC20250217005).

**FIGURE 1:**
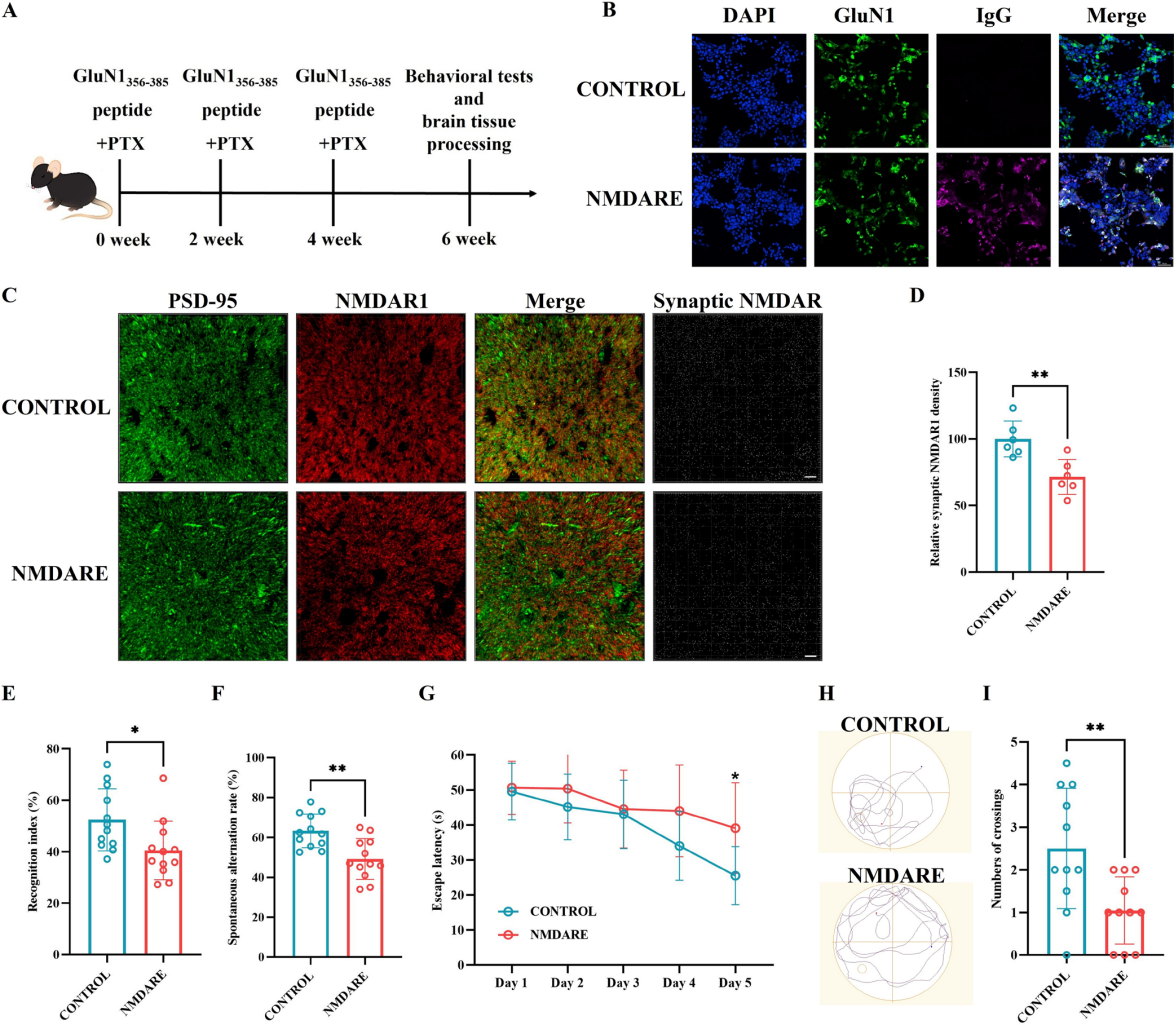
Establishment of an anti‐NMDAR encephalitis mouse model. (A) Schematic diagram of the experimental design. (B) Detection of serum anti‐NMDAR antibodies by cell‐based assay; scale bar = 50 μm. (C) Representative immunofluorescence images of the hippocampal CA3 region stained for PSD95 (green) and NMDAR1 (red); scale bar = 10 μm. (D) Quantitative analysis of synaptic NMDAR puncta; *N* = 6. (E) Recognition index in the novel object recognition test; *N* = 12. (F) Spontaneous alternation rate in the Y‐maze test; *N* = 12. (G) Escape latency in the Morris water maze test; *N* = 12. (H) Representative swimming trajectories during probe trials. (I) Number of target platform crossings in the Morris water maze test; *N* = 12. Comparisons were performed using Student's t test. *p < 0.05, **p < 0.005.

To induce a mouse model of anti‐NMDAR encephalitis, we adopted an active immunization strategy with slight modifications to the protocol previously described [26]. Specifically, mice were subcutaneously immunized with 200 μg of the GluN1_356–385_ peptide (LQNRKLVQVGIYNGTHVIPNDRKIIWPGGE, GL Biochem, China), emulsified in Complete Freund's Adjuvant (CFA, F5881, Sigma‐Aldrich, USA) containing *Mycobacterium tuberculosis* H37Ra (4 mg/mL; 231,141, BD DIFCO, USA), resulting in a final peptide concentration of 1 mg/mL. Two booster immunizations were administered at 2 and 4 weeks following the initial injection. Mice in the control group received an equivalent volume of phosphate‐buffered saline emulsified with CFA. In addition, all animals were administered pertussis toxin intraperitoneally (200 ng; 180, List Biological Laboratories, USA) on the day of the first immunization and again 48 h later. A series of behavioral tests and brain tissue procedures were performed 6 weeks after the initial immunization (Figure 1A).

### 2.5 Proteomic Analysis

Hippocampal proteomic analysis was performed by Bioprofile (Shanghai, China) using data‐independent acquisition (DIA) mass spectrometry. Briefly, hippocampal tissue samples were collected and lysed for protein extraction. Extracted proteins were digested with trypsin to generate peptides for LC–MS/MS analysis. Peptides from each sample were separated by liquid chromatography using a Vanquish Neo UHPLC system (Thermo Scientific). After chromatographic separation, peptides were analyzed by DIA on an Orbitrap Astral mass spectrometer (Thermo Scientific). Raw mass spectrometry data were processed using DIA‐NN software for spectral library‐free identification and protein quantification. Protein identification was performed against the UniProt *Mus musculus* reference proteome (TaxID:10090, 88,473 entries, release date: 2023‐07‐17). Differentially expressed proteins (DEP) were defined as those with a | LogFC | > 1 and a *p*‐value < 0.05.

### 2.6 Western Blotting

Total protein was isolated from hippocampus using a commercial extraction kit (BB‐3101‐100 T, BestBio, China). Protein concentrations were determined using the bicinchoninic acid assay (23225, Thermo Fisher, USA). Equal amounts of protein (30 μg) were separated by SDS‐polyacrylamide gel electrophoresis and subsequently transferred onto polyvinylidene fluoride membranes. Membranes were blocked in 5% non‐fat milk solution and incubated with the following primary antibodies overnight at 4°C: rabbit polyclonal anti‐CHI3L1 antibody (1:1000; 12036‐1‐AP, Proteintech, Wuhan, China), rabbit polyclonal anti‐CRTH2 antibody (1:1000; 251678, Zenbio, Chengdu, China), and rabbit monoclonal anti‐β‐actin antibody (1:50000; PSH03‐63, Huabio, Hangzhou, China). Following washing steps, membranes were incubated with horseradish peroxidase (HRP)‐conjugated secondary antibodies (1:5000; 511,203, Zenbio, Chengdu, China) for 1 h at room temperature. Signals were detected using an enhanced chemiluminescence system (iBright FL1500, Thermo Fisher, USA) and quantified using the iBright Analysis Software.

### 2.7 Immunofluorescence Staining

To assess synaptic NMDAR1 density, seven‐micrometer‐thick hippocampal coronal sections were prepared using a freezing microtome (Leica, Germany). Non‐permeabilized sections were first incubated overnight at 4°C with a rat monoclonal antibody against the extracellular domain of GluN1 (MAB10655, 1:100; R&D Systems, USA). Subsequently, sections were incubated at room temperature for 1 h with goat anti‐rat Alexa Fluor 555 secondary antibody (ab150158, 1:500; Abcam, UK). Following this, the sections were permeabilized with 0.3% Triton X‐100 for 30 min at room temperature and then sequentially incubated with rabbit polyclonal anti‐PSD‐95 antibody (ab18258, 1:500; Abcam), goat anti‐rabbit Alexa Fluor 488 (A‐11008, 1:1000; Invitrogen), and DAPI (DA001; LEAGENE, China) for 15 min each at room temperature. After staining, the slides were mounted using ProLong Gold antifade reagent (P10144; Invitrogen). Images were acquired using a confocal microscope as previously described. Quantification of NMDAR and PSD‐95 puncta, as well as their colocalization, was performed using the spot detection algorithm in Imaris Suite 9.0 (Oxford Instruments). Postsynaptic NMDAR clusters were defined as NMDAR puncta colocalized within 0.2 μm of PSD‐95 puncta [27].

For detection of CHI3L1 expression and neurogenesis, coronal brain sections (30 μm thick) were prepared using a cryostat for subsequent immunofluorescence staining. To minimize nonspecific antibody binding, tissue sections were blocked at room temperature for 1 h in a solution containing 5% goat serum, 5% bovine serum albumin, and 0.3% Triton X‐100. After blocking, the slices were incubated overnight at 4°C with the following primary antibodies: rabbit polyclonal anti‐CHI3L1 antibody (1:200; 12036‐1‐AP, Proteintech), mouse monoclonal anti‐IBA1 antibody (1:500; RT1316, Huabio), mouse monoclonal anti‐GFAP antibody (1:500; EM140707, Huabio), rabbit monoclonal anti‐doublecortin (DCX) antibody (1:500; ET1701‐98, Huabio), and rat monoclonal anti‐SOX2 antibody (1:500; HA601396, Huabio). The next day, goat anti‐mouse Alexa Fluor 555 (A‐21422, 1:1000, Invitrogen), goat anti‐rabbit Alexa Fluor 488 (A‐11008, 1:1000, Invitrogen), or goat anti‐rat Alexa Fluor 555 secondary antibody (ab150158, 1:500; Abcam) were applied for 1 h at room temperature, followed by counterstaining with DAPI for 15 min. Finally, sections were mounted using ProLong Gold Antifade Mountant and visualized using a Nikon AX confocal microscope (Nikon, Japan).

### 2.8 Statistical Analysis

Quantitative data are expressed as mean ± standard deviation (SD). Data normality was assessed using the Shapiro–Wilk test (α = 0.05). For descriptive comparisons between groups, normally distributed continuous variables were analyzed using unpaired two‐tailed Student's *t*‐tests, whereas non‐normally distributed variables were compared using the Mann–Whitney *U* test. Categorical variables were compared using the *χ* ^2^ test or Fisher's exact test, as appropriate.

To evaluate the independent association between CHI3L1 levels and 1‐year outcomes, multivariable logistic regression models were constructed. CHI3L1 levels were modeled as continuous predictors after log10 transformation and *z*‐score standardization, and effect estimates are reported per 1‐SD increase. Variables showing *p* < 0.10 in univariable comparisons were included as covariates in the multivariable models. Discriminative performance of serum and CSF CHI3L1 for predicting 1‐year cognitive complaints was evaluated by receiver operating characteristic (ROC) curve analysis. The optimal cut‐off values for CHI3L1 were determined using the Youden index. Based on these thresholds, patients were stratified into High‐CHI3L1 and Low‐CHI3L1 groups for additional comparisons of baseline clinical characteristics and longitudinal outcomes. The flow of patients from baseline CHI3L1 status to 1‐year functional and cognitive outcomes was visualized using Sankey diagrams.

All statistical analyses were conducted using GraphPad Prism 9 (GraphPad Software, USA) and R software (version 4.5.1). A P value less than 0.05 was considered statistically significant. The specific statistical tests used for each dataset are indicated in the corresponding figure legends.

## 3 Results

### 3.1 Establishment of an Anti‐NMDAR Encephalitis Mouse Model

As illustrated in Figure 1, immunization with the GluN1_356–385_ peptide effectively induced the production of anti‐NMDAR antibodies in mice (Figure 1B). Immunofluorescence analysis further revealed a significant reduction in synaptic NMDAR1 density in the hippocampus of these mice (Figure 1C,D). Behaviorally, mice with anti‐NMDAR encephalitis (NMDARE) exhibited marked cognitive deficits, as demonstrated by a lower discrimination index in the novel object recognition test (34.02% vs. 55.41%, *p* = 0.0006; Figure 1E) and a reduced spontaneous alternation rate in the Y‐maze test (55.21% vs. 63.76%, *p* = 0.0085; Figure 1F), compared to control mice. In addition, NMDARE mice exhibited a significantly prolonged escape latency (39.10 s vs. 25.52 s, *p* = 0.0321) and a reduced number of target platform crossings (1.05 vs. 2.50, *p* = 0.0074) in the Morris water maze test (Figure 1G–I). These findings are consistent with the neuropathological alterations and cognitive dysfunction commonly observed in patients with anti‐NMDAR encephalitis.

### 3.2 Upregulation of CHI3L1 in the Hippocampus of Anti‐NMDAR Encephalitis Mice

**FIGURE 2:**
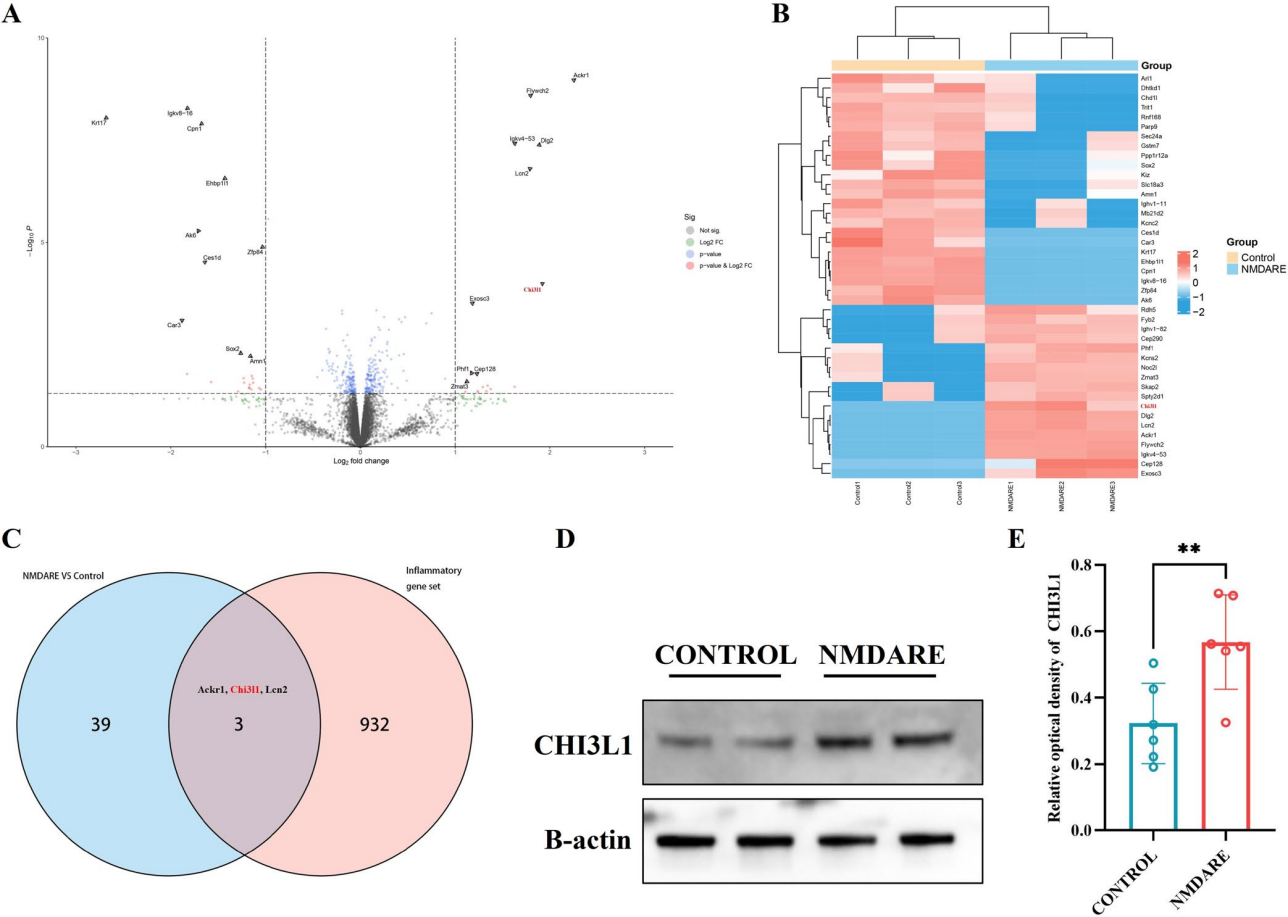
Upregulation of CHI3L1 in the Hippocampus of Anti‐NMDAR Encephalitis Mice. (A) Volcano plot showing differentially expressed proteins (DEPs). (B) Heatmap illustrating the expression patterns of 42 DEPs across samples. (C) Venn diagram showing the overlap between DEPs and a predefined inflammation‐related protein set, identifying 3 overlapping proteins. (D) Representative Western blot images of hippocampal CHI3L1 expression. (E) Quantification of hippocampal CHI3L1 protein levels; *N* = 6. Comparisons were performed using Student's *t*‐test. **p < 0.005.

Based on the proteomic analysis, a total of 42 differentially expressed proteins (DEPs) were identified, including 18 upregulated and 24 downregulated proteins. A volcano plot illustrates the distribution of these DEPs, with the top 10 most significantly upregulated and downregulated proteins highlighted (Figure 2A). A heatmap further visualizes the expression profiles of these 42 proteins across individual samples (Figure 2B). To explore the potential involvement of inflammatory processes, these DEPs were intersected with a predefined inflammation‐related gene set, yielding three overlapping candidates: Ackr1, Chi3l1, and Lcn2 (Figure 2C). Considering the relevance of Chi3l1 reported in two previous clinical studies of anti‐NMDAR encephalitis [23, 24], this protein was selected for further investigation. Subsequent Western blot analysis confirmed a significant upregulation of Chi3l1 protein levels (0.57 vs. 0.32, *p* = 0.0092) in the hippocampus of NMDARE mice compared to controls (Figure 2D,E).

### 3.3 CHI3L1 Is Predominantly Expressed in GFAP^+^ Astrocytes

**FIGURE 3:**
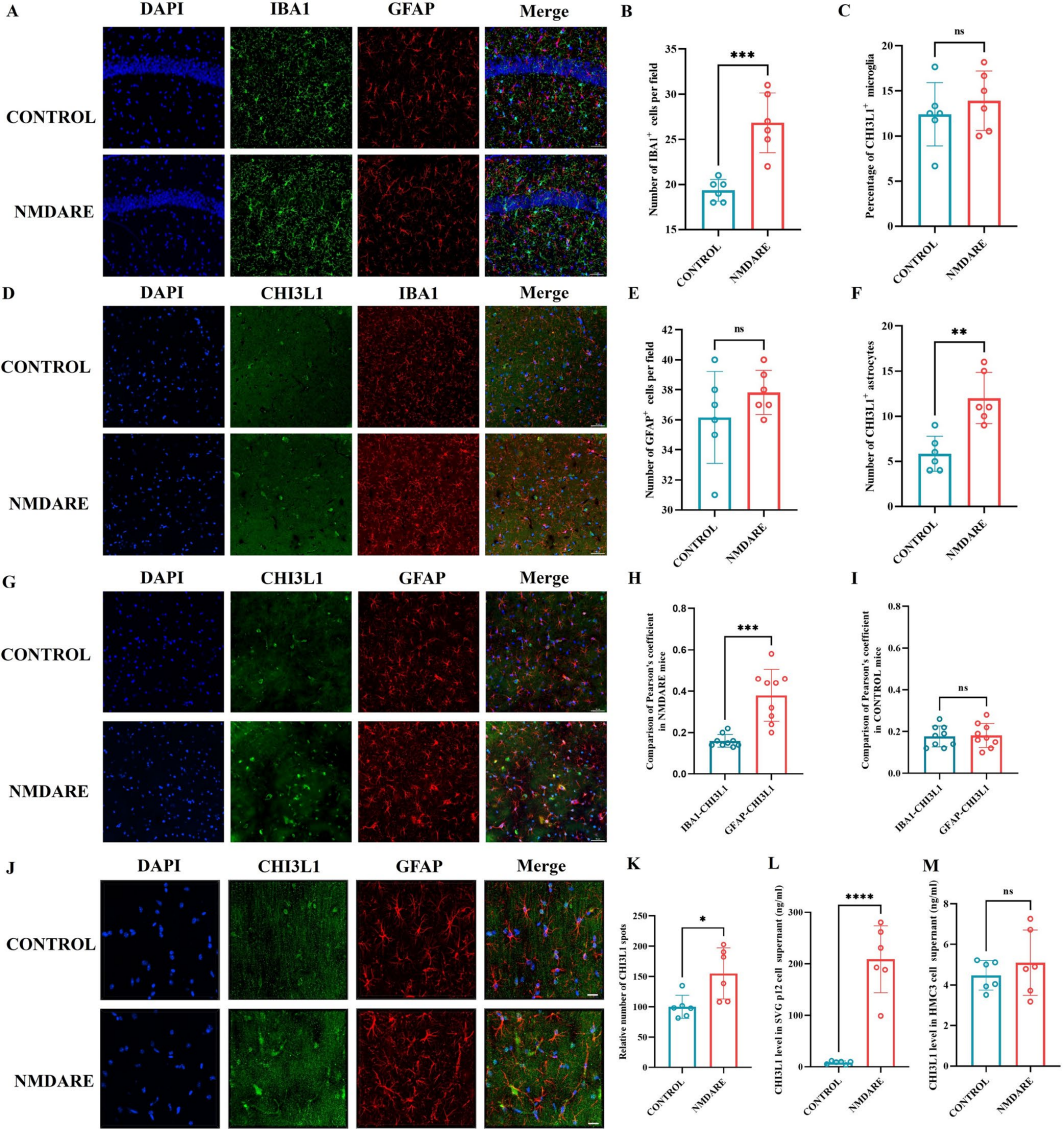
CHI3L1 Is Predominantly Expressed in GFAP^+^ Astrocytes. (A) Representative immunofluorescence images of the hippocampal CA1 region stained for IBA1 and GFAP; scale bar = 50 μm. (B) Quantification of IBA1^+^ cell numbers; *N* = 6. (C) Quantification of GFAP^+^ cell numbers; *N* = 6. (D) Representative immunofluorescence images of the hippocampal CA3 region stained for CHI3L1 and IBA1; scale bar = 50 μm. (E) Quantification of the percentage of CHI3L1^+^ cells among IBA1^+^ cells; *N* = 6. (F) Quantification of CHI3L1^+^ cells among GFAP^+^ cells; *N* = 6. (G) Representative immunofluorescence images of the hippocampal CA3 region stained for CHI3L1 and GFAP; scale bar = 50 μm. (H) Comparison of Pearson's correlation coefficient in NMDARE mice. (I) Comparison of Pearson's correlation coefficient in Control mice. (J) High‐magnification immunofluorescence images of the hippocampal CA3 region stained for CHI3L1 and GFAP; scale bar = 10 μm. (K) Quantification of CHI3L1^+^ puncta; *N* = 6. (L) Measurement of CHI3L1 level in human astrocyte cell line SVG p12 supernatants; *N* = 6. (M) Measurement of CHI3L1 level in human microglial cell line HMC3 supernatants; *N* = 6. Comparisons were performed using Student's *t*‐test. *p < 0.05, **p < 0.005, ***p < 0.0005, ****p < 0.0001, ns, not significant.

We first evaluated glial activation in the hippocampus of NMDARE mice using immunofluorescence staining (Figure 3A). Quantitative analysis demonstrated a significant increase in IBA1‐positive microglia compared with controls (26.83 vs. 19.33, *p* = 0.0004; Figure 3B), whereas the number of GFAP‐positive astrocytes remained statistically unchanged (37.83 vs. 36.17, *p* = 0.2570; Figure 3C). To determine the cellular source of CHI3L1, we performed co‐immunostaining for CHI3L1 with IBA1 or GFAP. Results showed a comparable proportion of CHI3L1 co‐localized with IBA1‐positive microglia in NMDARE and control mice (13.90% vs. 12.40%, *p* = 0.4633; Figure 3D,E). In contrast, CHI3L1 co‐localization with GFAP‐positive astrocytes was significantly increased in NMDARE mice (12.00% vs. 5.83%, *p* = 0.0013; Figure 3F,G). Consistently, the Pearson's correlation coefficient for CHI3L1–GFAP was markedly higher than that for CHI3L1–IBA1 in NMDARE mice (0.38 vs. 0.16, *p* = 0.0001; Figure 3H), whereas no such difference was observed in controls (0.18 vs. 0.17, *p* = 0.8636; Figure 3I), further supporting astrocytes as the principal CHI3L1‐expressing cell population. Although the correlation coefficients differed significantly, their absolute values remained relatively low. Given that CHI3L1 is a secreted protein, we further assessed its overall abundance by performing spot analysis using Imaris software. A significant elevation in CHI3L1‐positive puncta was observed in the hippocampus of NMDARE mice relative to controls (155.3% vs. 100.0%, *p* = 0.0151; Figure 3J,K). To further validate the glial origin of CHI3L1, we stimulated the human astrocyte cell line SVG p12 and the human microglial cell line HMC3 with pooled CSF from patients with anti‐NMDAR encephalitis or controls and measured CHI3L1 levels in culture supernatants. Astrocytes exhibited a robust increase in CHI3L1 secretion following patient's CSF stimulation (209.00 vs. 8.32 ng/mL, *p* < 0.001; Figure 3L), whereas microglia did not show a significant change (4.469 vs. 5.094 ng/mL, *p* = 0.407; Figure 3M). Together, these findings demonstrate that hippocampal CHI3L1 levels are markedly elevated in NMDARE mice and indicate that astrocytes—rather than microglia—represent the predominant source of increased CHI3L1 expression and secretion.

### 3.4 Impaired Hippocampal Neurogenesis in Anti‐NMDAR Encephalitis Mice

**FIGURE 4:**
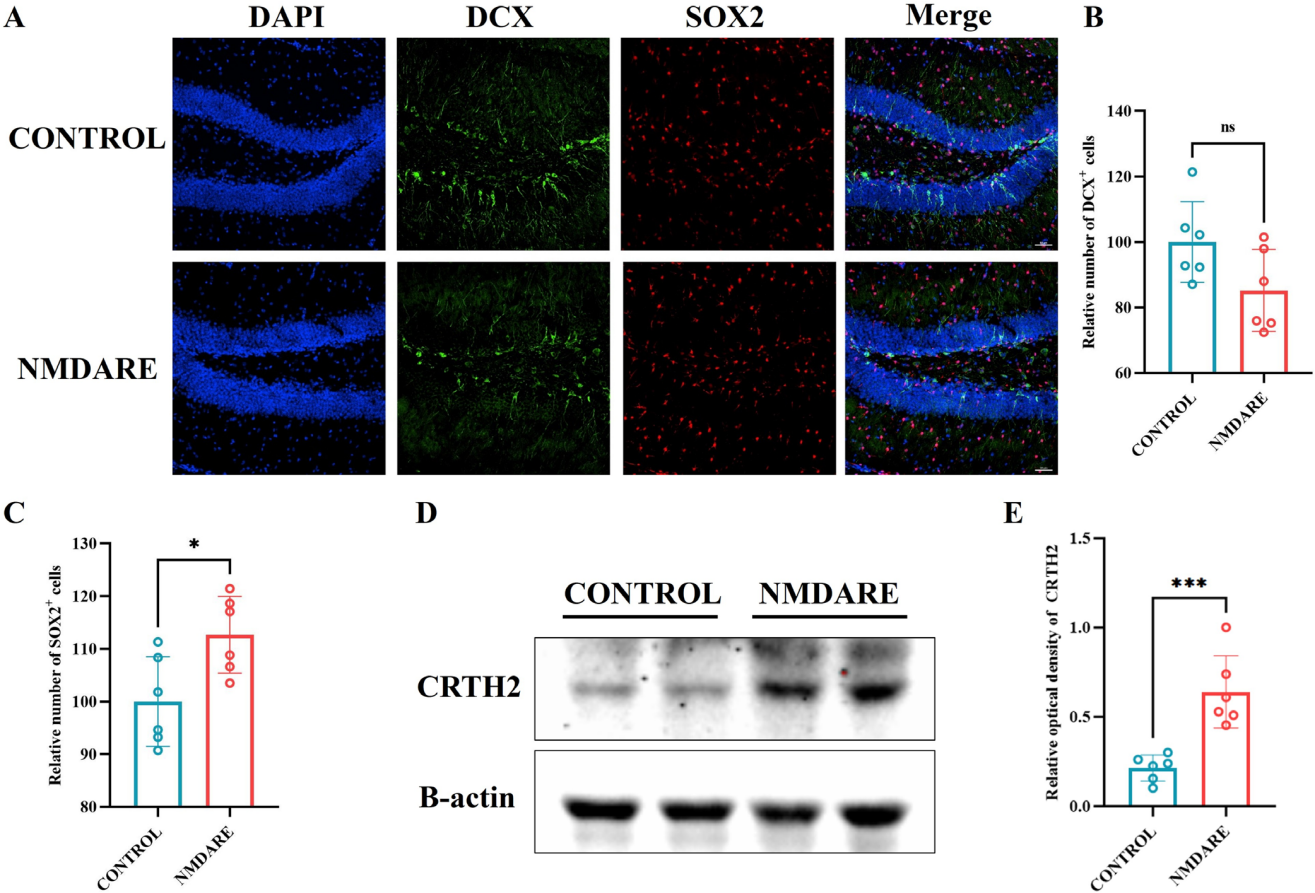
Impaired Hippocampal Neurogenesis in Anti‐NMDAR Encephalitis Mice. (A) Representative immunofluorescence images of the hippocampal dentate gyrus (DG) region stained for DCX and SOX2; scale bar = 50 μm. (B) Quantification of DCX^+^ cell numbers; *N* = 6. (C) Quantification of SOX2^+^ cell numbers; *N* = 6. (D) Representative Western blot images of hippocampal CRTH2 expression. (E) Quantification of hippocampal CRTH2 protein levels; *N* = 6. Comparisons were performed using Student's t test. ns, not significant, *p < 0.05, ***p < 0.0005.

To further explore potential mechanisms underlying long‐term cognitive impairment in patients with anti‐NMDAR encephalitis, we assessed hippocampal neurogenesis, as previous studies have shown that CHI3L1 can impair neural stem cell (NSC) differentiation through CRTH2‐mediated suppression of β‐catenin signaling [19, 28]. As shown in Figure 4A, NMDARE mice exhibited a trend toward reduced numbers of DCX‐positive immature neurons in the dentate gyrus compared to controls, although this difference did not reach statistical significance (85.2% vs. 100.0%, *p* = 0.0657; Figure 4B). In contrast, the number of SOX2‐positive neural stem/progenitor cells was significantly increased in NMDARE mice relative to controls (122.7% vs. 100.0%, *p* = 0.0198; Figure 4C), suggesting a potential disruption in the transition from progenitors to newly generated neurons. Moreover, the expression of CRTH2 in the hippocampus of NMDARE mice was significantly higher than that of controls (0.64 vs. 0.21, *p* = 0.0007; Figure 4D,E). These findings indicate dysregulated hippocampal neurogenesis and support the possibility that CHI3L1‐CRTH2 signaling contributes to impaired neurogenesis in NMDARE mice.

### 3.5 Elevated CHI3L1 Levels in Children With Anti‐NMDAR Encephalitis

A total of 83 pediatric patients diagnosed with anti‐NMDAR encephalitis were included, with a mean age of 8.45 ± 3.98 years and 73.5% being female. A detailed summary of their clinical characteristics is provided in Table 1.

**TABLE 1:** Clinical characteristics of children with anti‐NMDAR encephalitis.

Characteristics	Patients (*n*, %)
Gender (female)	61 (73.5)
Age (years, mean)	8.45 ± 3.98
Major symptoms	
Psychiatric behavior/cognitive dysfunction	81 (97.6)
Seizures	55 (66.3)
Movement disorders	70 (84.3)
Speech dysfunction	64 (77.1)
Decreased consciousness	28 (33.7)
Autonomic dysfunction/central hypoventilation	30 (36.1)
Auxiliary examination results	
Abnormal EEG	69 (87.3)
Abnormal brain MRI	33 (40.7)
CSF WBC count (×106/L)	12.5 (4.75–32.0)
CSF protein (g/L)	0.27 (0.19–0.42)
Second‐line immunotherapy	20 (24.1)
Good outcome at 12 month	72 (88.9)
Cognitive complaints at 12 month	47 (58.0)

Abbreviations: CSF, cerebrospinal fluid; EEG, electroencephalogram; MRI, magnetic resonance imaging; NMDARE, anti‐N‐methyl‐D‐aspartate receptor encephalitis; WBC, white blood cell.

**FIGURE 5:**
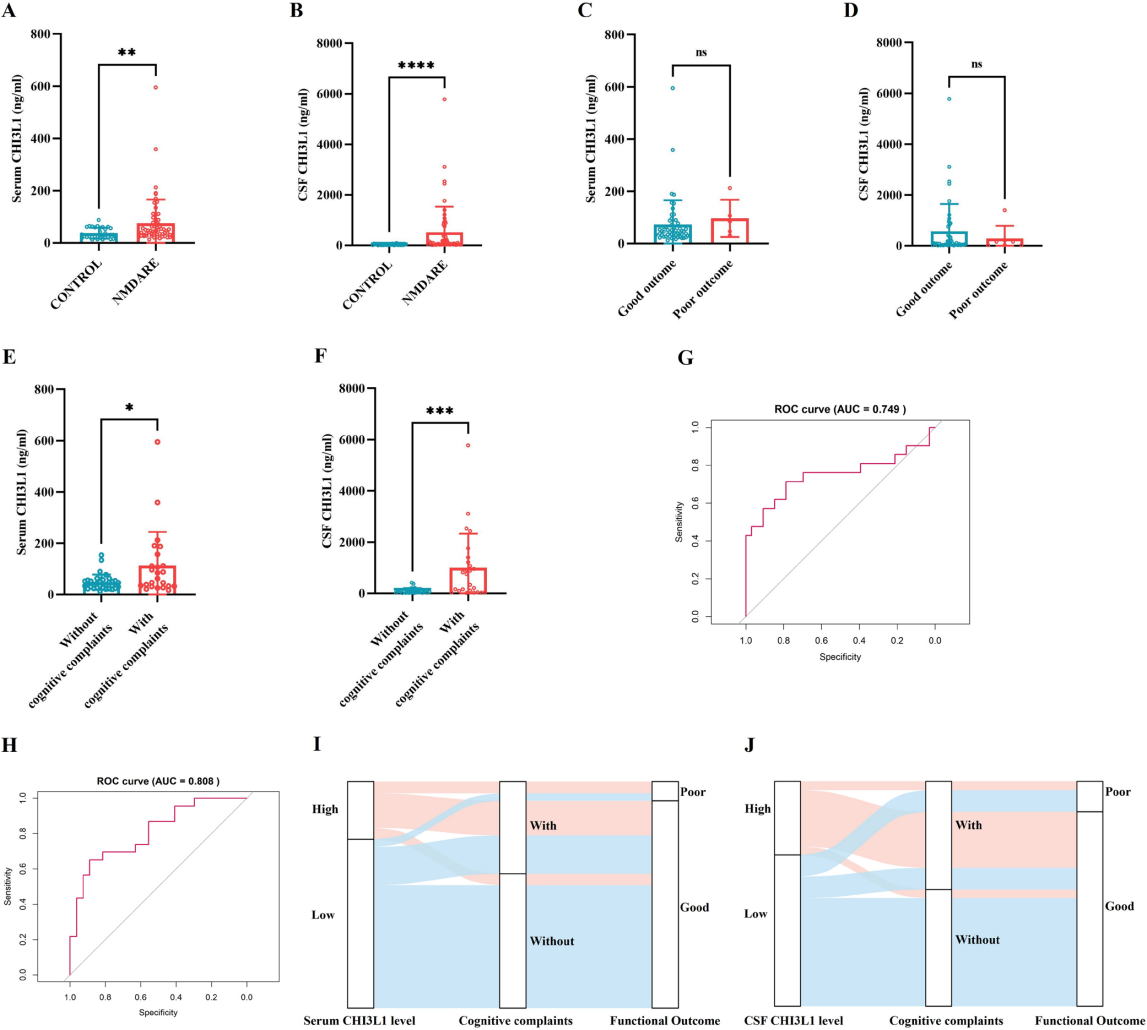
Elevated CHI3L1 Levels in Children with Anti‐NMDAR Encephalitis. (A) Quantification of serum CHI3L1 levels; Controls, *N* = 31; NMDARE, *N* = 61. (B) Quantification of CSF CHI3L1 levels; Controls, *N* = 31; NMDARE, *N* = 53. (C) Comparison of serum CHI3L1 levels between patients with good (*N* = 54) and poor (*N* = 5) outcomes. (D) Comparison of CSF CHI3L1 levels between patients with good (*N* = 44) and poor (*N* = 7) outcomes. (E) Comparison of serum CHI3L1 levels between patients with (*N* = 24) or without (*N* = 35) cognitive complaints. (F) Comparison of CSF CHI3L1 levels between patients with (*N* = 25) or without (*N* = 27) cognitive complaints. (G) ROC curve showing the discriminative performance of serum CHI3L1 in predicting 1‐year cognitive complaints. (H) ROC curve showing the discriminative performance of CSF CHI3L1 in predicting 1‐year cognitive complaints. (I) Sankey diagram illustrating the association between baseline serum CHI3L1 levels and 1‐year outcomes. (J) Sankey diagram illustrating the association between baseline CSF CHI3L1 levels and 1‐year outcomes. Comparisons were performed using Mann–Whitney *U* test. *p < 0.05, **p < 0.005, ***p < 0.0005, ****p < 0.0001, ns, not significant.

Serum CHI3L1 levels were measured in 61 patients, showing a median level of 45.94 ng/mL (IQR: 30.00–84.38 ng/mL), which was significantly elevated compared to controls who had a median level of 30.42 ng/mL (IQR: 20.52–58.25 ng/mL) (*p* = 0.0055; Figure 5A). Similarly, CSF CHI3L1 levels were assessed in 53 patients and found to have a median level of 87.41 ng/mL (IQR: 39.30–417.14 ng/mL), significantly higher than the median CSF level in controls at 30.42 ng/mL (IQR: 17.55–35.99 ng/mL) (*p* < 0.0001; Figure 5B).

### 3.6 Elevated CHI3L1 Levels Are Associated With Poor Cognitive Outcomes in Children With Anti‐NMDAR Encephalitis

Of the 83 enrolled patients, two were lost to follow‐up, leaving 81 individuals who completed the 1‐year follow‐up assessment. Based on the mRS scores at 1 year, patients were classified into good and poor outcome groups. No significant differences in serum or CSF CHI3L1 levels were observed between these two groups (Figure 5C,D). Significant between‐group differences were observed in sex distribution, decreased consciousness, abnormal MRI findings, and peak mRS score (Table S1). After adjustment for these covariates, neither serum nor CSF CHI3L1 levels showed an independent association with functional outcome. However, when specifically analyzing cognitive outcomes, serum CHI3L1 levels were significantly lower in patients with complete cognitive recovery (*n* = 35) compared to those who reported cognitive complaints at the 1‐year follow‐up (*n* = 24) (40.59 vs. 73.47 ng/mL, *p* = 0.0369; Figure 5E). Consistently, CSF CHI3L1 levels were markedly higher in patients with cognitive complaints (*n* = 25) than in those with complete recovery (*n* = 27) (754.33 vs. 48.21 ng/mL, *p* = 0.0004; Figure 5F). Notably, children with persistent cognitive impairment at 1 year tended to present with decreased consciousness during the acute phase more frequently than those who fully recovered (44.1% vs. 25.5%, *p* = 0.080; Table S2). We therefore proceeded to multivariable logistic regression to determine whether CHI3L1 levels were independently associated with cognitive outcomes. In multivariable logistic models, serum CHI3L1 (OR: 2.57, 95% CI 1.38–5.47, *p* = 0.006; Table S3) and CSF CHI3L1 (OR: 4.28, 95% CI 1.97–11.49, *p* = 0.001; Table S4) levels remained independently associated with cognitive impairment. ROC analyses further demonstrated good discrimination (serum AUC = 0.749, 95% CI 0.592–0.906; CSF AUC = 0.808, 95% CI 0.688–0.929; Figure 5G,H). Optimal cut‐off values derived from the Youden index were 80.88 pg/mL for serum CHI3L1 and 204.72 pg/mL for CSF CHI3L1. Based on these thresholds, 25.4% (15/59) of patients were classified as High‐CHI3L1 for serum and 32.7% (17/52) as High‐CHI3L1 for CSF. The longitudinal trajectory of patients from baseline High/Low‐CHI3L1 groups to 1‐year cognitive and functional outcomes is illustrated using Sankey diagrams (Figure 5I,J). Clinical characteristics of the High and Low CHI3L1 groups are summarized in Table 2. Consistent with the multivariable regression results, both serum High‐CHI3L1 (80.0% vs. 27.3%, *p* < 0.001) and CSF High‐CHI3L1 (88.2% vs. 28.6%, *p* < 0.001) groups exhibited substantially higher rates of persistent cognitive complaints at 1 year compared with their respective Low‐CHI3L1 groups, whereas no significant differences were observed in overall functional outcomes.

**TABLE 2:** Comparison of clinical characteristics between patients with high and low CHI3L1 levels in Serum or CSF.

	Serum		*p*	CSF		*p*
	Low (*n* = 44)	High (*n* = 15)		Low (*n* = 35)	High (*n* = 17)	
Gender (female)	35 (79.5)	7 (46.7)	0.036^a^	23 (65.7)	13 (76.5)	0.430
Age (years, median)	8.21 (5.02–11.61)	9.67 (4.75–12.17)	0.449	9.92 (7.92–12.25)	4.75 (2.79–10.79)	0.027
Major symptoms (*n*, %)						
Psychiatric behavior/cognitive dysfunction	43 (97.7)	15 (100.0)	1.000^b^	33 (94.3)	17 (100.0)	1.000^b^
Seizures	30 (68.2)	9 (60.0)	0.563	20 (57.1)	12 (70.6)	0.350
Movement disorders	37 (84.1)	11 (73.3)	0.589^a^	31 (88.6)	14 (82.4)	0.855^a^
Speech dysfunction	32 (72.7)	12 (80.0)	0.830^a^	27 (77.1)	13 (76.5)	1.000^a^
Decreased consciousness	14 (31.8)	6 (40.0)	0.563	13 (37.1)	4 (23.5)	0.326
Autonomic dysfunction/central hypoventilation	17 (38.6)	4 (26.7)	0.403	15 (42.9)	5 (29.4)	0.350
Auxiliary examination results (*n*, %)						
Abnormal EEG	10 (25.0)	5 (33.3)	0.781^a^	19 (54.3)	7 (46.7)	0.621
Abnormal brain MRI	17 (40.5)	7 (46.7)	0.677	16 (45.7)	6 (35.3)	0.476
Leukocytosis and/or elevated protein in CSF	20 (46.5)	6 (40.0)	0.662	19 (54.3)	9 (52.9)	0.927
Subsequent immunotherapy	20 (45.5)	4 (26.7)	0.201	13 (37.1)	5 (29.4)	0.583
mRS at peak	4.0 (3.0–5.0)	3.0 (3.0–5.0)	0.847	4.0 (3.0–5.0)	3.0 (3.0–5.0)	0.467
Poor functional outcome	2 (4.5)	3 (20.0)	0.187^a^	5 (14.3)	2 (11.8)	1.000^a^
With cognitive complaints	12 (27.3)	12 (80.0)	< 0.001	10 (28.6)	15 (88.2)	< 0.001

Abbreviations: CSF, cerebrospinal fluid; EEG, Electroencephalogram; MRI, magnetic resonance imaging; mRS, modified Ranking Scale; WBC, white blood cell.

^a^ *χ* ^2^ correction for continuity.

^b^ Fisher's exact test.

Taken together, these findings suggest that elevated CHI3L1 levels are more closely associated with long‐term cognitive impairment than with general functional disability, supporting the potential of CHI3L1 as a clinically meaningful biomarker for predicting cognitive outcomes in pediatric anti‐NMDAR encephalitis.

## 4 Discussion

In this study, we first established an active immunization mouse model of anti‐NMDAR encephalitis. Using proteomic analysis combined with a series of molecular and histological experiments, we demonstrated a significant upregulation of CHI3L1 in the hippocampus of NMDARE mice. Importantly, our findings provide preliminary direct evidence that astrocytes play a major role in the elevation of CHI3L1 in the context of anti‐NMDAR encephalitis. Building upon these experimental findings, we further confirmed, for the first time, that both serum and CSF CHI3L1 levels are significantly elevated in a pediatric cohort of patients with anti‐NMDAR encephalitis. Moreover, elevated CHI3L1 levels were closely associated with long‐term cognitive impairment. Together, these results suggest that CHI3L1 may serve as a valuable biomarker for cognitive dysfunction in anti‐NMDAR encephalitis.

Anti‐NMDAR encephalitis is the most common form of autoimmune encephalitis, with a rapidly increasing incidence worldwide. It accounts for approximately 70%–80% (50%–60% if MOG antibody‐associated cases are included) of pediatric cases with anti‐neuronal antibody‐mediated autoimmune encephalitis [29, 30, 31, 32]. The mRS is commonly used to assess general functional outcomes in these patients. Based on mRS evaluations, current first‐ and second‐line immunotherapies are generally considered effective, with only about 10%–20% of pediatric patients exhibiting poor outcomes [3, 33, 34, 35]. However, the mRS primarily reflects physical and daily functional status and may not adequately capture cognitive or behavioral deficits. Recent studies have raised concerns regarding long‐term cognitive outcomes in this population. For example, a French cohort study with at least 2 years of follow‐up reported that more than 45% of children with anti‐NMDAR encephalitis experienced cognitive and academic difficulties despite favorable mRS scores [13]. Another study with a minimum 5‐year follow‐up similarly showed that although most patients achieved substantial or full recovery based on conventional assessments, approximately one‐fifth still exhibited behavioral and academic impairments [16]. In line with these findings, our study demonstrated that while 88.9% of patients achieved favorable functional outcomes at the 1‐year follow‐up according to mRS, over half still reported cognitive complaints across different domains.

Multiple biomarkers have been investigated for their potential association with outcomes in anti‐NMDAR encephalitis [36, 37, 38], yet most studies have primarily focused on their relationship with mRS scores. A recent study reported that elevated serum or plasma NfL levels were significantly associated with unfavorable recovery and severe cognitive impairment at 2‐year follow‐up in children with anti‐NMDAR encephalitis [15]. However, other studies have suggested that the association between NfL levels and mRS‐based outcomes remains confounded or ambiguous, with age being a major contributing factor [39]. These results highlight the limitations of conventional outcome measures and emphasize the need for reliable biomarkers and mechanistic research to better evaluate and address cognitive sequelae in anti‐NMDAR encephalitis.

CHI3L1 is a secreted glycoprotein belonging to the glycoside hydrolase family 18. Although it lacks enzymatic chitinase activity, CHI3L1 is involved in various biological processes, particularly the regulation of inflammatory responses. In the CNS, CHI3L1 is predominantly produced by reactive astrocytes and microglia under pathological conditions, where it plays a key role in modulating neuroinflammation. Specifically, CHI3L1 has been shown to influence glial activation, promote the release of pro‐inflammatory cytokines, and contribute to extracellular matrix remodeling, thus participating in both tissue repair and disease progression [17, 22, 40, 41]. Two previous studies have explored CHI3L1 levels in adult anti‐NMDAR encephalitis cohorts [23, 24]. While both studies demonstrated elevated CHI3L1 levels, the findings regarding its association with mRS‐defined outcomes were inconsistent. In the present study, we extended these observations to a large pediatric cohort and, for the first time, comprehensively evaluated CHI3L1 levels in both serum and CSF. Notably, we found that although CHI3L1 levels were not associated with mRS scores at 1‐year follow‐up, they were significantly correlated with cognitive impairment. These findings suggest that, beyond the existing biomarkers reflecting general functional outcomes, CHI3L1 may serve as a novel biomarker for predicting long‐term cognitive sequelae in patients with anti‐NMDAR encephalitis.

Although NMDARs on neuronal membranes are the direct targets of pathogenic antibodies in anti‐NMDAR encephalitis, accumulating evidence from brain autopsy studies has demonstrated prominent glial activation in affected patients [42]. In particular, the involvement of microglia in the pathogenesis of anti‐NMDAR encephalitis has been confirmed in various animal models [43, 44] and in vitro experiments [45]. However, the role of astrocytes in this context remains poorly understood. Previous studies reported no significant changes in the overall number of GFAP‐positive astrocytes in the hippocampus of anti‐NMDAR encephalitis mice [46, 47]. In the present study, through a combination of proteomic analysis, molecular assays, and histological experiments, we demonstrated that CHI3L1 expression is significantly upregulated during the course of anti‐NMDAR encephalitis. More importantly, using in vivo fluorescence colocalization together with in vitro stimulation of different glial cell types, we identified astrocytes as the predominant source of this elevation. These findings suggest that, although the overall number of astrocytes remains relatively unchanged, their functional state is altered in this disease context. Specifically, reactive astrocytes may contribute to the neuroinflammatory process at least in part through increased secretion of CHI3L1. This provides novel insights into the previously underappreciated role of astrocytes in the pathogenesis of anti‐NMDAR encephalitis.

Although the internalization of membrane NMDARs induced by anti‐NMDAR antibodies has been shown to be reversible [48, 49], the mechanisms underlying antibody‐induced cognitive impairment, as well as the potential involvement of CHI3L1, remain largely unclear. A recent study by Taraschenko et al. provides important clues in this regard. Their work demonstrated that mice infused with patient‐derived anti‐NMDAR antibodies exhibited persistent memory deficits even after antibody clearance. Histological analysis further revealed reduced numbers of DCX positive immature neurons, increased ectopic localization of Prox1 positive granule cells in the dentate hilus, and an abnormal ratio of low‐ to fast‐proliferating BrdU‐labeled cells in the hippocampus [50]. In our study, although BrdU or EdU labeling was not performed, we similarly observed an increased number of SOX2 positive neural progenitor cells and a mild reduction of DCX positive immature neurons within the dentate gyrus. These findings indicate disrupted hippocampal neurogenesis in NMDARE mice, characterized by impaired differentiation of neural progenitor cells, reduced maturation of newborn neurons, and aberrant neuronal migration, which may collectively contribute to sustained cognitive impairment.

Interestingly, recent studies in other autoimmune‐mediated neuroinflammatory disorders, such as neuromyelitis optica and multiple sclerosis, have shed light on the potential role of CHI3L1 in regulating hippocampal neurogenesis and cognitive function. In both disease models, astrocyte‐derived CHI3L1 was found to suppress Sox2^+^ progenitor proliferation and reduced DCX^+^ immature neurons via activation of the CRTH2/β‐catenin signaling pathway. Moreover, CHI3L1 disrupted dendritic development and synaptic integrity, exacerbating neurogenic deficits. Importantly, genetic deletion or pharmacological blockade of CHI3L1 in these models restored neurogenesis and improved cognitive performance [19, 28]. More recently, a study in Alzheimer's disease provided even more direct evidence by using human iPSC‐derived neural progenitor cells, demonstrating that CHI3L1 suppresses neuronal differentiation and β‐catenin activity in a CRTH2‐dependent manner [51]. These findings collectively suggest a potentially shared pathogenic mechanism across neuroimmune diseases, whereby astrocyte‐derived CHI3L1 contributes to hippocampal neurogenic dysfunction and cognitive decline. In light of these findings, our results show that CHI3L1 expression is markedly elevated in anti‐NMDAR encephalitis, accompanied by signs of disrupted hippocampal neurogenesis and a significant upregulation of CRTH2 in the hippocampus. Together, these observations raise the possibility that a similar astrocyte‐derived CHI3L1–CRTH2 signaling axis may contribute to impaired neurogenesis and long‐term cognitive deficits in anti‐NMDAR encephalitis, warranting further mechanistic investigation.

The present study has several limitations. First, although we demonstrated that CHI3L1 is markedly elevated in anti‐NMDAR encephalitis and observed accompanying abnormalities in hippocampal neurogenesis, we did not establish a direct causal relationship between CHI3L1 and impaired neurogenesis at the cellular level. Due to the inability to obtain reliable human neural stem cell lines, we were unable to perform in vitro neurogenesis assays to determine whether CHI3L1 directly suppresses neuronal differentiation, as reported in MS, NMOSD, and Alzheimer's disease models. Meanwhile, the evaluation of hippocampal neurogenesis in our animal experiments remains relatively preliminary. We primarily assessed SOX2 and DCX expression and did not include proliferative markers such as EdU/BrdU or Ki67, nor co‐labeling strategies (e.g., SOX2–GFAP to identify radial glia–like NSC) that could more precisely delineate the specific stages of neurogenic disruption. A more comprehensive neurogenic profiling will be required to fully define how CHI3L1 dysregulates the NSC–neuroblast transition in anti‐NMDAR encephalitis. Second, we did not perform interventional experiments (such as genetic deletion and pharmacological blockade of CHI3L1 or CRTH2) to determine whether targeting this pathway can restore neurogenesis or improve cognitive outcomes. Such strategies may offer novel therapeutic insights for mitigating long‐term neurological sequelae in affected patients. Third, cognitive outcomes in this study were primarily based on subjective reports obtained during routine clinical follow‐up, without the use of standardized neuropsychological assessment tools. This limitation may affect the reliability of the associations observed. Further validation in well‐designed prospective clinical cohorts with comprehensive and objective cognitive evaluations will be essential to confirm our findings.

## 5 Conclusion

Our study provides preliminary evidence that CHI3L1 is elevated during the course of anti‐NMDAR encephalitis, suggesting a potential role of astrocyte‐mediated neuroinflammation in impaired neurogenesis. These findings indicate that CHI3L1 may serve as a promising biomarker and therapeutic target for cognitive dysfunction associated with this disease.

## Author Contributions

Conceptualization, L.J., J.M., and H.L.; methodology, Y.L., R.D., H.L., and J.Y.; validation, H.L. and Y.L.; formal analysis, Y.L., R.D., and J.Y.; investigation, J.Y. and Y.L.; resources, X.Y., Z.H., X.L, J.L., Y.J., and L.C.; writing – original draft preparation, H.L.; writing – review and editing, all authors; visualization, Y.L., J.Y., and H.L.; supervision, J.M., and L.J.; project administration, H.L.; funding acquisition, H.L. and L.J. All authors have read and agreed to the published version of the manuscript.

## Funding

This work was supported by the National Natural Science Foundation of China, 82501626. Natural Science Foundation of Chongqing, China, CSTB2024NSCQ‐MSX0506. Chongqing medical scientific research project (Joint project of Chongqing Health Commission and Science and Technology Bureau), 2025QNXM010.

## Ethics Statement

All animal protocols were approved by the Ethics Committee of the Children's Hospital of Chongqing Medical University (Approval No. CHCMU‐IACUC20250217005). Patient recruitment and sample collection were approved by the Institutional Review Board of the Children's Hospital of Chongqing Medical University (Approval No. 2024344).

## Conflicts of Interest

The authors declare no conflicts of interest.

## Supporting information


**Table S1:** Clinical characteristics of patients with different prognosis at 1 year of disease course.
**Table S2:** Clinical characteristics of two groups of patients with or without cognitive complaints at 1 year of disease course.
**Table S3:** Logistic regression analysis of transformed serum CHI3L1 levels as an independent predictor of 1‐year cognitive outcomes.
**Table S4:** Logistic regression analysis of transformed CSF CHI3L1 levels as an independent predictor of 1‐year cognitive outcomes.

## Data Availability

The data that support the findings of this study are available from the corresponding author upon reasonable request.
