# Supplementary material for: Elevated CHI3L1 as a Potential Biomarker of Cognitive Dysfunction in Anti‐NMDAR Encephalitis: Evidence From Clinical Results and Mice Model
Source: CNS Neurosci Ther. 2026 Jan 5;32(1):e70739. doi: 10.1002/cns.70739 (PMC12766899; doi:10.1002/cns.70739)
Supplement: Supplementary file 1 — Table S1: Clinical characteristics of patients with different prognosis at 1 year of disease course. Table S2: Clinical characteristics of two groups of patients with or without cognitive complaints at 1 year of disease course. Table S3: Logistic regression analysis of transformed serum CHI3L1 levels as an independent predictor of 1‐year cognitive outcomes. Table S4: Logistic regression analysis of transformed CSF CHI3L1 levels as an independent predictor of 1‐year cognitive outcomes. [file CNS-32-e70739-s001.docx]

**Elevated CHI3L1 as a Potential Biomarker of** **Cognitive Dysfunction in Anti-NMDAR Encephalitis: Evidence from Clinical results and Mice Model**

Yuhang Li^1†^, Ran Ding^1†^, Jiaxin Yang^1^, Xiaoyue Yang^1^, Ziyao Han^1^, Xue Li^1^, Jie Liu^1^, Yan Jiang^1^, Li Cheng^1^, Jiannan Ma^1^, Hanyu Luo^1*^, Li Jiang^1*^

Supplementary table 1 Clinical characteristics of patients with different prognosis at 1 year of disease course

|  | Good outcome  (n=72) | Poor outcome  (n=9) | *P* value |
| --- | --- | --- | --- |
| Gender(female) | 53(73.6) | 6(66.7) | 0.002^b^ |
| Age (years, median) | 8.80(5.21-11.61) | 4.33(2.17-13.5) | 0.367 |
| **Major symptoms (n**, **%)** |  |  |  |
| Psychiatric behavior/cognitive dysfunction | 70(97.2) | 9(100.0) | 1.000^c^ |
| Seizures | 47(65.3) | 7(77.8) | 0.708^b^ |
| Movement disorders | 59(81.9) | 9(100.0) | 0.363^b^ |
| Speech dysfunction | 53(73.6) | 9(100.0) | 0.179^b^ |
| Decreased consciousness | 19(26.4) | 8(29.6) | 0.001^b^ |
| Autonomic dysfunction/ central hypoventilation | 24(33.3) | 6(66.7) | 0.113^b^ |
| **Auxiliary examination results** |  |  |  |
| Abnormal EEG (n=79） | 30(44.1) | 3(33.3) | 0.798^b^ |
| Abnormal brain MRI (n=77） | 25(35.7) | 8(88.9) | 0.007^b^ |
| Leukocytosis and/or elevated protein in CSF (n=80) | 33(46.5) | 4(44.4) | 1.000^b^ |
| Subsequent immunotherapy | 28(38.9) | 6(66.7) | 0.217^b^ |
| mRS at peak |  |  | <0.001 |
| Serum CHI3L1 level (ng/ml)(n=59) | 44.74(329.08-873.08) | 84.38(38.69-159.59) | 0.203 |
| CSF CHI3L1 level (ng/ml)(n=52) | 187.41(41.02-784.72) | 154.51(25.34-205.84) | 0.753 |

Abbreviations: EEG = Electroencephalogram; MRI = magnetic resonance imaging; CSF = cerebrospinal fluid; WBC = white blood cell; mRS = modified Ranking Scale.

^b^ χ2 correction for continuity; ^c^ Fisher’s exact test.

Supplementary table 2 Clinical characteristics of two groups of patients with or without cognitive complaints at 1 year of disease course

|  | Without cognitive complaints (n=47) | With cognitive complaints (n=34) | *P* value |
| --- | --- | --- | --- |
| Gender(female) | 35(74.5) | 24(70.6) | 0.698 |
| Age (years, median) | 8.92(5.58-11.42) | 7.75(3.84-12.19) | 0.455 |
| **Major symptoms (n**, **%)** |  |  |  |
| Psychiatric behavior/cognitive dysfunction | 45(95.7) | 34(100.0) | 0.507^c^ |
| Seizures | 29(61.7) | 25(73.5) | 0.256 |
| Movement disorders | 38(80.9) | 30(88.2) | 0.372 |
| Speech dysfunction | 34(72.3) | 28(82.4) | 0.294 |
| Decreased consciousness | 12(25.5) | 15(44.1) | 0.080 |
| Autonomic dysfunction/ central hypoventilation | 15(31,9) | 15(44.1) | 0.262 |
| **Auxiliary examination results** |  |  |  |
| Abnormal EEG (n=79） | 21(45.7) | 12(38.7) | 0.546 |
| Abnormal brain MRI (n=77） | 17(37.8) | 16(47.1) | 0.408 |
| Leukocytosis and/or elevated protein in CSF (n=80) | 20(43.5) | 17(50.0) | 0.563 |
| Subsequent immunotherapy | 19(40.4) | 15(44.1) | 0.740 |
| mRS at peak |  |  | 0.249 |
| Serum CHI3L1 level (ng/ml)(n=59) | 40.59 | 73.47 | 0.037 |
| CSF CHI3L1 level (ng/ml)(n=52) | 48.21 | 754.33 | 0.004 |

Abbreviations: EEG = Electroencephalogram; MRI = magnetic resonance imaging; CSF = cerebrospinal fluid; WBC = white blood cell; mRS = modified Ranking Scale.

^b^ χ2 correction for continuity; ^c^ Fisher’s exact test.

Supplementary table 3 Logistic regression analysis of transformed serum CHI3L1 levels as an independent predictor of 1-year cognitive outcomes

|  | β | OR | 95% CI | P |
| --- | --- | --- | --- | --- |
| Transformed Serum CHI3L1 level | 0.943 | 2.5688 | 1.383-5.475 | 0.006 |
| Decreased consciousness | 1.252 | 3.4988 | 0.982-13.653 | 0.059 |

Supplementary table 4 Logistic regression analysis of transformed CSF CHI3L1 levels as an independent predictor of 1-year cognitive outcomes

|  | β | OR | 95% CI | P |
| --- | --- | --- | --- | --- |
| Transformed CSF CHI3L1 level | 1.4545 | 4.2822 | 1.9701-11.486 | 0.001 |
| Decreased consciousness | 1.2338 | 3.4344 | 0.828-16.424 | 0.100 |
